# Spectral responses in rangelands and land cover change by livestock in regions of the Caatinga biome, Brazil

**DOI:** 10.1038/s41598-021-97784-5

**Published:** 2021-09-14

**Authors:** Leonardo Fiusa de Morais, Ana Clara Rodrigues Cavalcante, Deodato do Nascimento Aquino, Felipe Hermínio Meireles Nogueira, Magno José Duarte Cândido

**Affiliations:** 1 Federal University of Ceará, Fortaleza, 60440-900 Brazil; 2 Brazilian Agricultural Research Corporation, Sobral, 62010-970 Brazil; 3 National Institute of Colonization and Agrarian Reform, Fortaleza, 60442-800 Brazil

**Keywords:** Plant sciences, Environmental sciences

## Abstract

This study aimed to analyze fragments of rangelands through spectral responses and land cover change by livestock in regions of the Caatinga biome through remote sensing. For spectral behavior, the surface reflectance bidirectional (SRB) and spectral indexes of vegetation were used to verify the ragelands seasonality. Land cover change detection of Ouricuri and Tauá through Landsat-8 images with a 16-day revisit interval, were processed in the Google Earth Engine platform (GEE) and software Quantum GIS version 2.18 (QGIS). In the GEE platform, annual mosaics and stacking of the spectral bands were generated for the classification of images, and in sequence the production of thematic maps in QGIS. The analysis of land cover change considered the classes: thinned Caatinga, conserved Caatinga, herbaceous vegetation, bare soil, water and others. The analysis of the spectral responses showed that the vegetation monitored in Ouricuri presented higher SRB in the infrared band and lower SRB in the red and blue bands, and that caused the pasture to produce higher vegetation indexes than the other locations. Through validation, it was observed that in Tauá, there was an overall accuracy of 91% and Kappa index of 89%, and in Ouricuri there was an overall accuracy of 90% and Kappa index of 86%, indicating excellent correctness of the classification model. The classification model proved to be effective in verifying the temporal and spatial land cover change, making it possible to identify places with the vegetation that was most affected and susceptible to degradation and generation of political support to minimize damage to the Caatinga Biome.

## Introduction

The Brazilian semiarid region is mostly located in a depression, with the predominance of stable air masses, and it has a unique environment in which rainfall is difficult to occur^1^. In this Brazilian region, the predominant vegetation is the Caatinga, presenting a great floristic diversity as the result of some managements of the vegetation^2^, which is composed of plants adapted to the dry climate.

The Caatinga is an exclusively Brazilian biome, and most of the management solutions for this biome consist of balancing economic production and development with conservation of the vegetation. Even though its use plays an important economic role for the country, the Caatinga biome region are often associated to drought and poverty. Therefore, livestock is an important source of income for the people who live in that region. The vegetation has been essentially used for rangeland purposes, and the use efficiency depends a lot on the condition of the forage supply to the animals^3^.

It is known that monitoring the dynamics of native vegetation is an indicative of management strategies and plans to obtain rational management and sustainability of the rangeland^4^. Thus, remote sensing tools have significant potential to monitor vegetation dynamics, and it allows the verification of events such as the beginning or peak of vegetation growth. Besides, another benefit of remote sensing is the possibility of evaluating large areas with good spatial resolution and low cost^5^.

The basic premise of the application of remote sensing to the assessment of vegetation is that differences or changes can be identified through variations in spectral responses, and this is due to the fact that the vegetation represents the reflection of radiation on Earth^6^. From this, spectral vegetation indexes have been frequently used for the evaluation of rangelands through remote sensing^7^. However, the choice of the most adequate vegetation index to represent the vegetation requires basic studies, which relate the variability of the structural conditions of the vegetation with the bidirectional reflectance factors of the surface of the different channels or spectral bands.

The satellite remote sensing platforms offer temporal image records, which enable studies on the change dynamics and changes in biomes over time. According to Midekisa^8^, quantifying and monitoring the spatial and temporal dynamics of land cover is essential for a better understanding of the cause of many processes that have resulted in unrecoverable changes in biomes. For a long time, the mapping of land cover change through remote sensing had computational limitations due to image processing^9^. However, the emergence of Google Earth Engine (GEE), a web platform with high computational capacity for storing cloud data and with a large catalog of images^10^, has made it possible to process and classify remote sensing images quickly^11^. Thus, the aim of this study was to characterize the spectral behavior, evaluating the seasonality of bidirectional surface reflectance (BSR) values and vegetation indexes of fragments of rangelands, as well as to evaluate the land cover change of different municipalities located in the Caatinga Biome in Brazil.

## Methods

### Studied area

**Figure 1:**
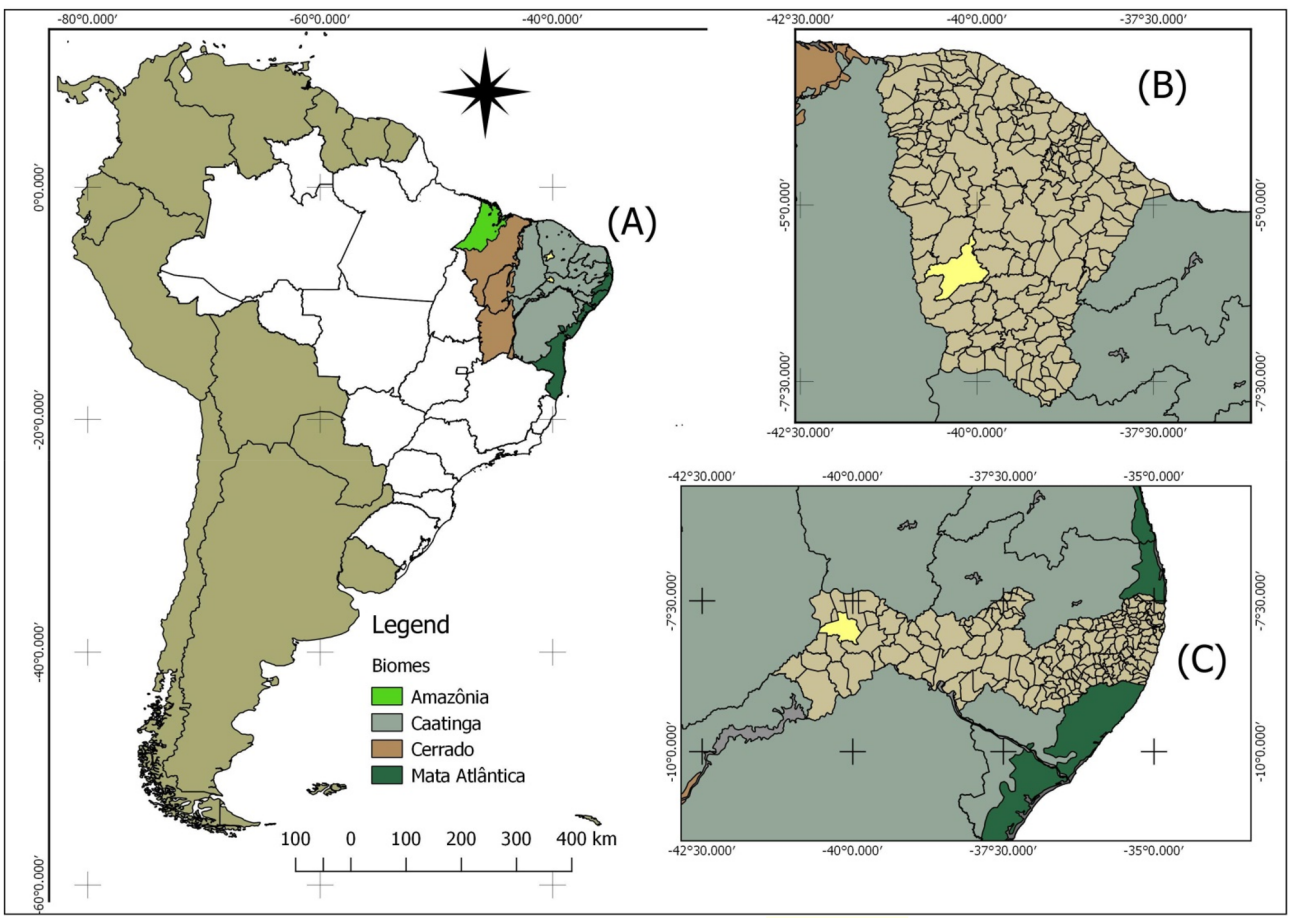
Biomes that are part of the Brazilian Northeast region (**A**), municipalities where permanent transects were established to assess the vegetation: Tauá (**B**) in the State of Ceará and Ouricuri (**C**) in the State of Pernambuco.

The field study was carried out on fragments of rangelands with different levels of woody density. Two fragments were established in the municipality of Tauá, located in the state of Ceará (Fig. 1B), and two others in the municipality of Ouricuri, state of Pernambuco (Fig. 1C), knowing that the two municipalities are located within the limits of the Caatinga Biome (Fig. 1A).

The choice of these municipalities was based on the regions that have important livestock activities, and the variability of climate, soil and structure of the Caatinga vegetation of these locations, being representative of the Caatinga rangelands. Three ecological sites were monitored, consisting of fragments of rangelands (with about 10 ha of area), two of which were located on the farm *Cachoeirinha do Pai Senhor* (Latitude − 5.62° S and Longitude − 40.12° W), at the Barra Nova district, in Tauá-CE, and the third one was located on the property of the Caatinga Association in Ouricuri-PE (Latitude − 7.97° S and Longitude − 40.15° W). Cross-shaped transects were established in areas with different density of woody plants, generating 4 quadrants of 25 m of length on each side from the center.

### Spectral responses of fragments of rangelands

After defining the woody density of the vegetation, the polygonal of the studied area was delimited, obtaining the limits through land control points using a GPS navigation device Garmim Etrex 10, and then processed in the Google Earth Pro tool. Images were obtained from the Landsat-8 satellite, which had an OLI sensor (Operational Land Imager). The images were obtained from the USGS (United States Geological Survey) on the Earth Explorer platform on the following dates: 05/28/2018 and 10/19/2018 (Tauá), and 05/28/2018 and 10/10/2018 (Ouricuri), noting that the images obtained in the month of May refer to the rainy season and those obtained in October to the dry season. The raw images went through the atmospheric correction procedure using the DOS (Dark Object Subtraction) method, proposed by Chavez^12^ and Chavez^13^, to obtain the surface reflectance factors through extension of the Semiautomatic Classification Plugin^14^ present in the QGIS software (2.18).

For the evaluation of the spectral behavior, a surface reflectance bidirectional (SRB) curve was generated for each fragment of rangeland during the rainy and dry seasons. For this, it was necessary to stack the bands, and extract the values contained in the pixels of the images: ρb2 (Blue), ρb3 (green), ρb4 (red), ρb5 (near infrared), b6 (SWIR 1; short-wave infrared 1) and ρb7 (SWIR 2; short-wave infrared 2), through the free software QGIS (version 2.18). The selection criterion of the pixel was through the creation of 50 random points within the polygon corresponding to the rangelands area, in which the surface reflectance values of each spectral band were sampled, and then the vegetation indexes were determined. The rangeland fragments used in this study are vegetation with an area of around 10 ha (100,000 m^2^). Each Landsat-8 pixel has 900 m^2^ (30 × 30 m), so in a fragment of rangeland with 111 pixels we sampled of 50 pixels. The surface reflectance values were used to obtain the spectral indexes of vegetation using the free software raster calculator tool QGIS (version 2.18). The spectral indexes of the vegetation used in this study and their respective formulas are listed in Table 1.

**Table 1:** Rangelands spectral indexes used in this study. ρb2-blue, ρb4-red, ρb5- infrared, ρb6-SWIR1. L, C1 and C2 are coefficients, where L = 0.5 to 0.25; C1 = 7.5; and C2 = 2.5.

Spectral indexes	Abbreviation	Formula	References
Normalized Difference Vegetation Index	NDVI	$\frac{{\left( {\rho b5 - \rho b4} \right)}}{{\left( {\rho b5 + \rho b4} \right)}}$	^15^
Soil Adjusted Vegetation Index	SAVI	$\frac{{\left( {1 + L} \right)*\left( {\rho b5 - \rho b4} \right)}}{{\left( {\rho 5 + \rho b4 + L} \right)}}$	^16^
Modified Soil-Adjusted Vegetation Index 2	mSAVI_2_	$\frac{{\left( {2*pb5 + 1 - \sqrt {\left( {2pb5 + 1} \right)^{2} - 8\left( {pb5 - pb4} \right)} } \right)}}{2}$	^17^
Normalized difference Water Index	NDWI	$\frac{{\left( {\rho b5 - \rho b6} \right)}}{{\left( {\rho b5 + \rho b6} \right)}}$	^18^
Enhanced Vegetation Index	EVI	$\frac{{2.5*\left( {\rho b5 - \rho b4} \right)}}{{\left( {1 + \rho b5 + C1*\rho b4 - C2*\rho b2} \right)}}$	^19^

In the application of SAVI, the soil adjustment coefficient varied: L = 0.5 was adopted for the thinner vegetation and L = 0.25 for the more conserved vegetation, since the thinned vegetation has a greater contribution to the soil element. The LAI (Leaf area index) consists of the following measure: leaf area/ground area (per m^2^), and for this study it was obtained from the model proposed by Bastiaanssen^20^ in Eq. 1.

1 $${\text{LAI}} = \frac{{ - ln\frac{{\left( {0.69 - Savi} \right)}}{0.59}}}{0.91}$$

NDVI was chosen for being an important index and that had already several applications in the Caatinga Biome, while other indexes were chosen for having soil and atmospheric correction factors, and the LAI for being an important index for handling rangelands. To verify the ability of the indexes to differentiate the locations studied and the seasonality of the vegetation, two groups were compared using non-parametric analysis with the application of the Wilcoxon t test for two samples considering a 5% significance level in the SAS Studio software. The spectral behavior was presented in graphs and the spectral indexes in box-plot graphs using the Sigma Plot software (version 11.0).

### Land cover change in regions of the Caatinga biome

To obtain the land cover change in Ouricuri and Tauá, images of the Landsat-8 OLI sensor were stored and processed on the Google Earth Engine platform (GEE). The GEE has a web programming interface and a collection of geospatial analytical tools, offering a model in spatial and temporal scales, which increases accessibility and guarantees users the possibility to access and perform procedures without limitations related to data storage capacity or computational processing^21^.

**Figure 2:**
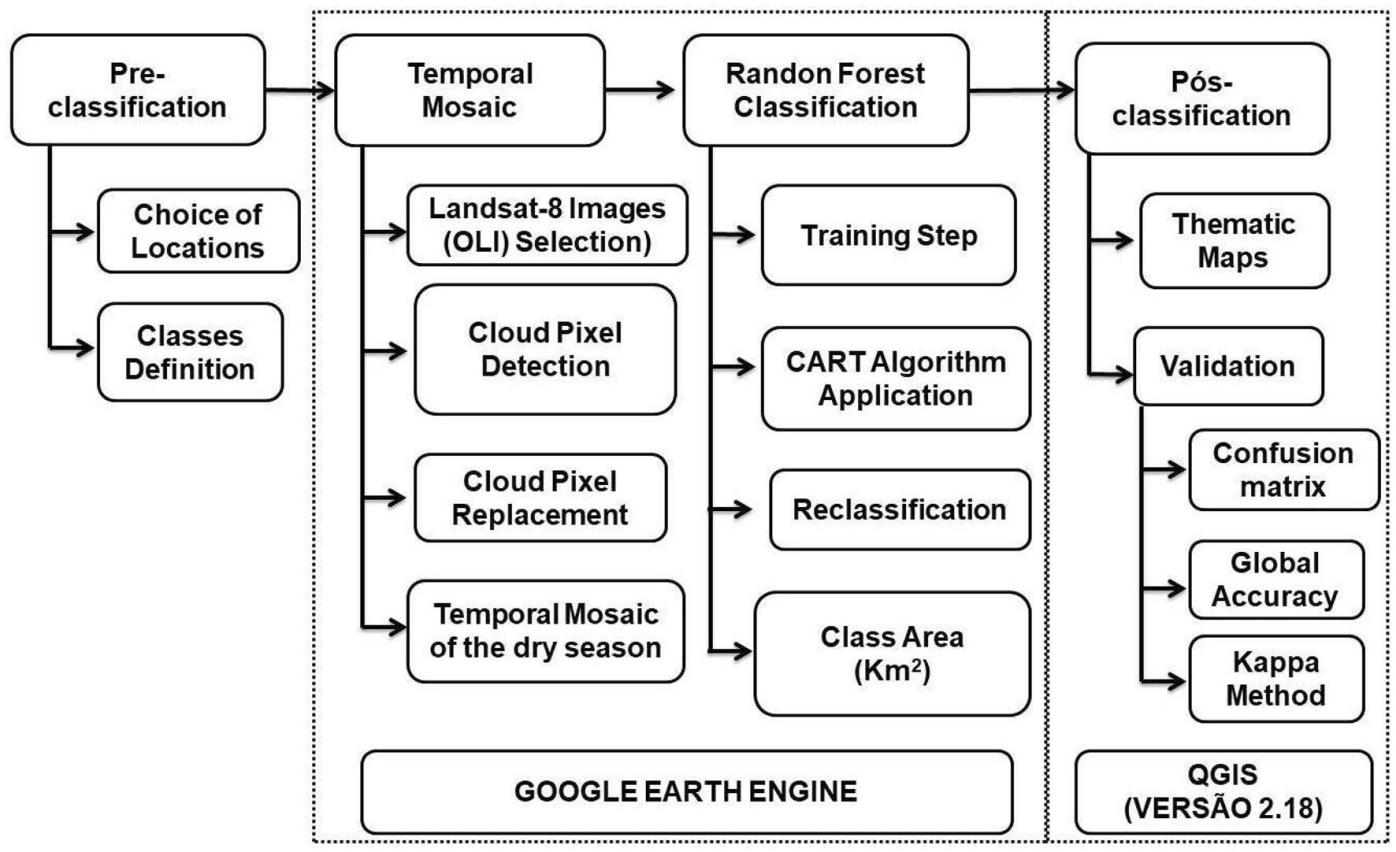
Model (Flowchart) of the steps taken to classify the land use cover.

The image processing consisted of two stages, one composed of image processing, which was performed on the Google Earth Engine platform itself, and the other one consisted of a map production and validation of the classification method in the free software Quantum GIS version 2.18 (Fig. 2). Initially, the raw scenes from Landsat 8 were processed to obtain the reflectance at the top of the atmosphere, and then they went through the process of removing the clouds and creating a temporal mosaic corresponding to the dry period of the year of study. During the removal of the clouds, it was used an algorithm that calculates the probability of the presence of a cloud in the range of zero to one hundred, by combining the brightness, temperature and NDSI (Normalized-Difference Snow Index) contained in the pixel. After identifying the pixel corresponding to the presence of a cloud, it was removed to make room for the pixel of the later available image, and the median of the pixels without the presence of clouds was calculated.

After the process of removing the clouds, a mosaic was generated corresponding to the composition of all scenes referring to the dry period of each year of study. As a visualization parameter, the RGB composition of Landsat 8 was used to highlight the NIR values (band 5) of the vegetation over the red tones of the image.

The generated annual compositions were used to carry out a supervised classification pixel by pixel, using the CART (Classification and Regression Trees) method, proposed by Breiman^22^. The CART classification method consists of an algorithm that checks the correlation of variables based on decision trees (Random Forest), where each tree consists of a new sample that is generated from the original data, and in each decision node the algorithm selects the more similar pixels in their respective classes^23^. After selecting points for training samples, the script is asked to process the data and provide the classification result, as well as the area in km^2^ of each class.

Table 2 shows in details the classes used in this study. The criterion for class definition was based on the interest of the present study to verify the temporal change of the Caatinga vegetation areas, considering that areas with predominance of herbaceous vegetation and thinning caatinga are used as rangelands. 990 pixels were obtained for each year considering the two studied sites. A total of 4950 pixels were sampled on the images corresponding to the locations during the 5 years of study, which were used for training the algorithm to identify the 6 classes (Table 2).

**Table 2:** Land cover classes used in this study and number of points obtained during the sampling training of the algorithm.

Class	Description	Points
Conserved Caatinga	Caatinga with dense vegetation and presence of deciduous tree plants with high biomass, with vegetation classified as Savannah-Stepic and Semideciduous Seasonal Forest^24^	180
Thinned Caatinga	Caatinga in secondary succession stage, with a shrubby-tree stratum consisting of a small density of species, characterized as a Woody Savannah-Stepic^24^	270
Herbaceous vegetation	Vegetation with predominance of herbaceous stratum without the presence of tree or shrub plants, characterized as Savannah-Stepic Grass-Woody vegetation^24^	150
Bare soil	Areas with varied coverage such as rocky outcrops, and other areas that prevail with exposed soil during the dry period of the year	200
Water	They include all of the features that contain water: reservoirs, rivers, streams, ponds, and others	150
Others	Urban areas, asphalt, civil construction areas and other infrastructures	40

After classification, the accuracy was evaluated through the application of confusion matrix, kappa index, errors of omission and commission. Samples were defined from high resolution images on Google Earth Pro, and then it was performed a visual interpretation check using the classified Landsat 8 image for the year 2018. The samples created were checked and defined as reference data (field truth), in order to build the confusion matrix later, and from this, obtain the accuracy and the coefficients of agreement with the QGIS software (2.18).

## Results

### Spectral responses of rangelands fragments

After obtaining the tree density, each transect received the name corresponding to the density of its vegetation: Ouricuri with the density of 292 plants/ha was called OD292; Tauá with the total density of 144 plants/ha was called TD144; and Tauá with the density of 280 plants/ha was called TD280.

**Figure 3:**
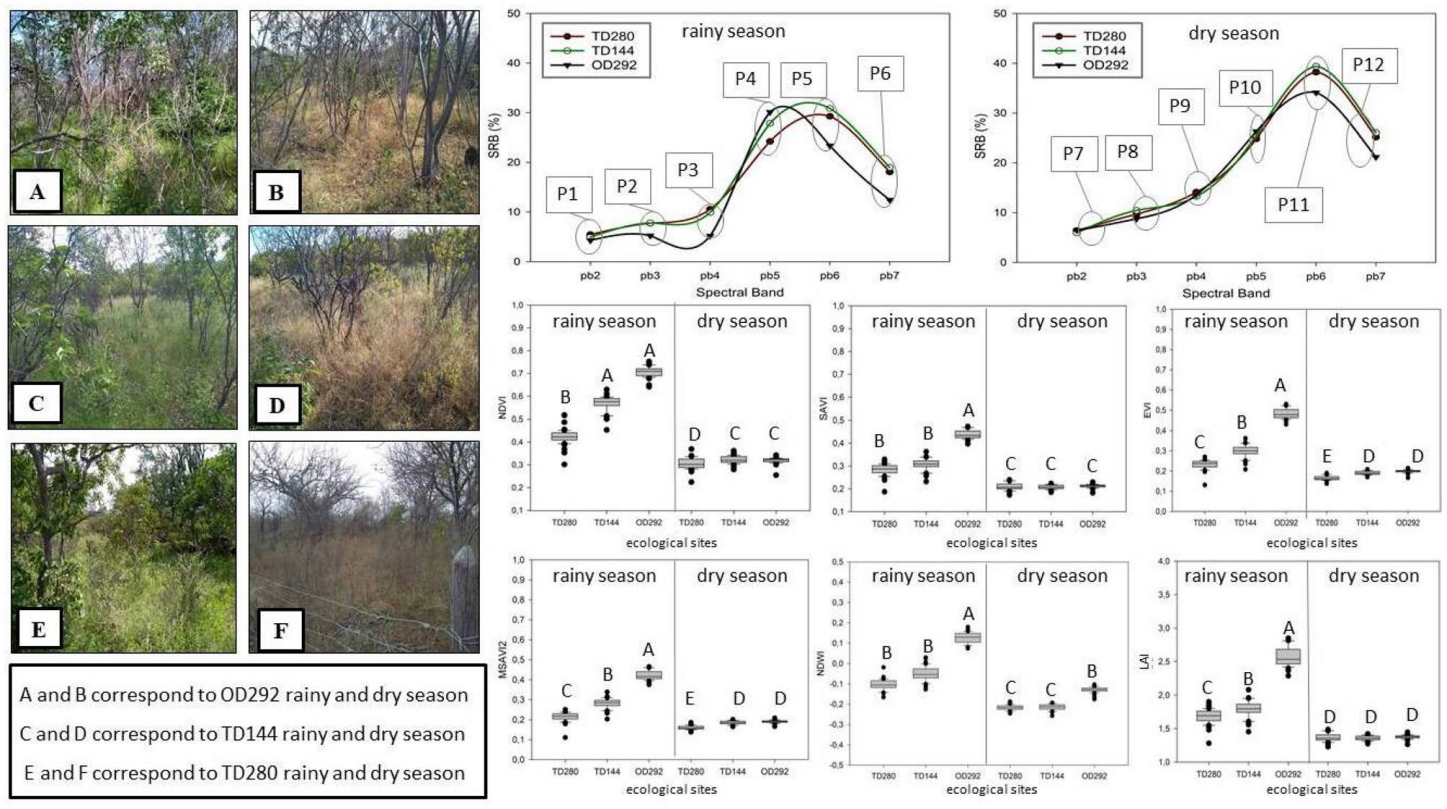
Surface reflectance bidirectional (SRB) and spectral indexes of rangelands with different densities of woody vegetation in Ouricuri and Tauá in the rainy and dry periods of the year 2018. Means followed by the same uppercase letter do not differ by the Wilcoxon t test at the level of significance of 5%.

Regardless of the monitoring location, a pattern of the spectral behavior of the rangeland was observed, presenting low SRB values of the spectral bands that correspond to blue, red, and green, and high SRB values of the infrared during the rainy season. Rangelands OD292 showed SRB values of the bands ρb2, ρb3, and ρb4 (visible wavelengths) of 4.2, 5.2, and 5.1%, respectively (Points P1, P2, P3 in Fig. 3), and the highest SRB in the ρb5 band (P4 in Fig. 3).

The rangelands located in Tauá had similar behavior regarding the SRB of the band ρb4, however, it was observed that Caatinga TD144 presented higher SRB values when compared to Caatinga TD280. Analyzing the dry period of the year, it was also observed a pattern of SRB of the different vegetations for the visible wavelengths, however, SRB was higher in the shortwave infrared region, which corresponds to the bands ρb6 and ρb7, (Fig. 3 point 11 and point 12) when compared to the rainy period (Fig. 3 point 5 and point 6). Observing the seasonal variation of the spectral vegetation indexes (Fig. 3), it was noted that all indexes presented higher values in vegetation OD292. Considering the rangelands monitored in Tauá (TD280 and TD144), the NDVI, EVI, mSAVI_2_, and LAI indexes were able to distinguish the rangeland during the rainy season, and the rangeland with the lowest woody density (TD144) presented the highest indexes, however, SAVI and NDWI were not able to differentiate the two rangelands. Comparing the seasons, and in the same rangeland, only SAVI and LAI did not show difference. EVI and mSAVI_2_ were the two most efficient indexes in distinguishing the vegetation, both considering the variation in woody density and seasonality of the rangeland.

### Land cover change in regions of the Caatinga biome

Both Tauá and Ouricuri showed increase in the area of conserved Caatinga and reduction in the area of thinned Caatinga (Table 3). In Tauá, the area of conserved Caatinga increased from 1423.7 km^2^ in 2014 to 2025.9 km^2^ in 2018, which represented an increase of 602 km^2^ or 42% of the conserved Caatinga, while in Ouricuri the area of conserved Caatinga went from 612.9 km^2^ in 2014 to 1059.2 km^2^ in 2018, representing an increase of 72.8%. In Tauá, the reduction of the area of thinned Caatinga and herbaceous vegetation was 33.9% and 58.3%, respectively, while there was an increase of 145% in the area of bare soil.

**Table 3:** Land cover change in Tauá and Ouricuri during the years 2014–2018.

Class	2014	2018	Change (km^2^)	Change (%)
	**Tauá**			
Thinned Caatinga	2155.8	1424.5	− 731.2	− 33.9
Conserved Caatinga	1423.7	2025.9	602.3	42.3
Herbaceous Vegetation	242.6	101.1	− 141.6	− 58.3
Bare Soil	176.4	432.9	256.5	145.3
Water	12.38	12.69	2.44	0.31
Others	18.78	20.31	4.3	22.9
	**Ouricuri**			
Thinned Caatinga	980.4	408.8	− 571.5	− 58.3
Conserved Caatinga	612.9	1059.2	446.3	72.8
Herbaceous Vegetation	123.2	26.8	− 96.3	− 78.17
Bare Soil	631.0	861.3	230.3	36.49
Water	6.0	6.7	0.71	11.79
Others	29.59	19.81	− 33.05	− 9.78

**Figure 4:**
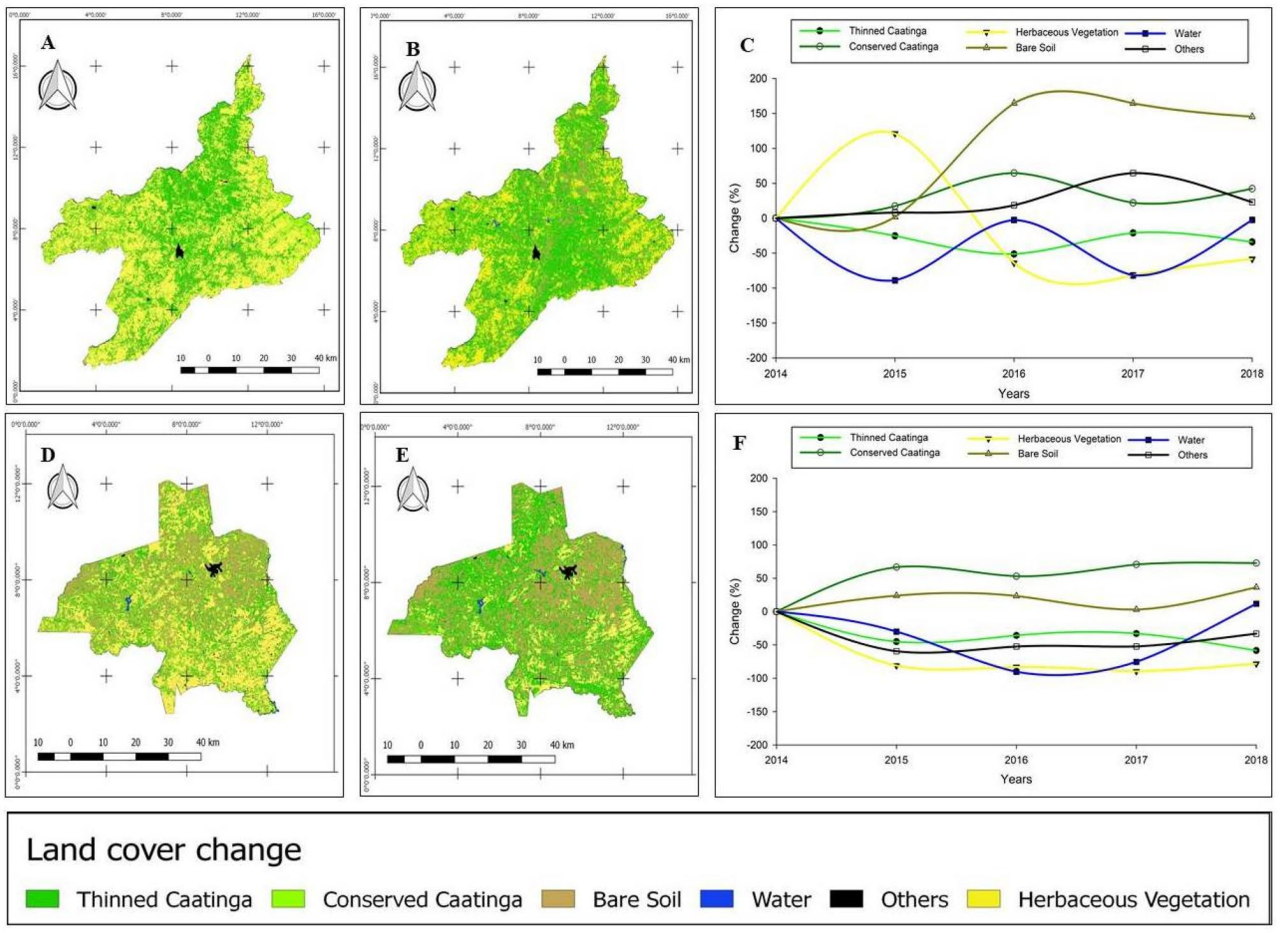
Land cover change and their respective classes obtained through image classification on Google Earth Engine of Tauá (**A**, **B**) and Ouricuri (**D**, **E**) for the years 2014 and 2018, and the interannual classes change (%) during the years of study (**C**–**F**).

In Ouricuri, there was reduction in the area of thinned caatinga and herbaceous vegetation of 58 and 78%, respectively, and an increase in the area of bare soil of 23%. Figure 4A, B, D, E are the maps of land cover change obtained in the years 2014 and 2018 respectively. It is visible in the maps the increase of the bare soil class, as well as the increase of the conserved caatinga class, when comparing the years of study. Figure 4C–F show the modifications of the classes analyzed over the studied period. In Tauá (Fig. 4C), there was an increase in the bare soil class from the year 2016, at the same time that there was a reduction in the classes of herbaceous vegetation and thinned caatinga. The water class increased in 2018 and in Tauá the smallest changes was in 2015. In both Ouricuri and Tauá, it was found that the largest areas corresponding to the water class were obtained in 2018, indicating that in that year the rainfall resulted in water supply to reservoirs in those municipalities.

Table 4 presents the confusion matrix comparing the classification on Google Earth Engine with the validation data observed. In Tauá, out of a total of 465 points established as field truth, 426 points agreed with the classification, which represents an overall accuracy of 91% and a Kappa index of 89%. In Ouricuri, out of the 468 points established as field truth, 423 points agreed with the classification, which resulted in an overall accuracy of 90% and a Kappa index of 86%. In both classifications of Tauá and Ouricuri, the water class showed the smallest errors of omission and commission, while the classes herbaceous vegetation and others showed the greatest errors of omission or commission.

**Table 4:** Confusion matrix, errors of omission and commission obtained by comparing the classification on Google Earth Engine (left) with the observed validation data (top) for the year 2018.

Class	Thinned	Conserved	Herbaceous	Bare Soil	Water	Others	Commission
	Tauá						
Thinned	68	2	3	0	0	5	0.16
Conserved	3	72	0	0	4	3	0.17
Herbaceous	2	0	91	0	3	0	0.20
Bare Soil	0	0	0	66	0	0	0.14
Water	0	0	0	0	4	0	0.86
Others	2	1	6	2	3	125	0.29
Omission	0.16	0.16	0.21	0.14	0.03	0.28	465
	Ouricuri						
Thinned	100	1	6	0	0	7	0.24
Conserved	0	20	2	0	0	5	0.05
Herbaceous	2	2	132	0	3	6	0.30
Bare Soil	0	0	0	40	0	0	0.08
Water	0	0	0	3	4	0	0.01
Others	3	0	12	9	1	127	0.32
Omission	0.22	0.04	0.32	0.11	0.01	0.30	468

## Discussion

### Spectral responses of fragments of rangelands

Lower SRB values of the wavelengths corresponding to the visible light during the dry period of the year are due to the characteristic of the Caatinga biome rangelands of presenting changes in their morphology throughout the year, and this causes variations in the fractions of absorbed, transmitted and reflected incident solar radiation. Comparing the different locations, it is important to note that there was a difference in the SRB of the vegetation, quite possibly due to the fact that Ouricuri had greater rainfall in 2018, in comparison to Tauá, which may have provided more abundant and vigorous vegetation, resulting in greater absorption of the visible wavelengths.

The knowledge of the spectral behavior of Caatinga rangelands is highly important since it is through these vegetation reflectance processes that it is possible to interpret the remote sensing of the vegetation, so that the contrast of the vegetation response in different wavelengths causes it to be highlighted in relation to the other targets, facilitating its identification and monitoring. According to Hatfield^25^, the understanding of leaf reflectance is important when the proposal is to monitor the vegetation by remote sensing methods, and it was from this understanding that it became possible to create several indexes to estimate parameters of the vegetation, such as leaf area, vegetation cover, biomass, vegetation type, and pasture productivity. In addition, the SRB pattern of vegetation makes it possible to assess the photosynthetic potential of the vegetation, the presence or not of pigments, water content, vegetation cover, and the presence of senescent material^26^.

In the Caatinga there is a large number of deciduous plants, which suffer direct effects from water availability^27^. Therefore, the lower spectral indexes of vegetation during the dry period of the year in detriment of the rainy period, are due to the lesser rainfall in the second half of 2018, and in response to water scarcity, the vegetation loses its foliage to reduce the loss of water through perspiration. Therefore, the higher values of spectral indexes in the rainy season occurred due to the greater vigor of the vegetation, due to the greater amount of photosynthetic pigments, which are found mainly in photosynthetically active leaves.

The average NDVI values were always higher than the other indexes analyzed in this study, during both rainy and dry periods. NDVI has been one of the most useful indexes, as it measures changes in the chlorophyll content, through the absorption of red radiation, and changes in the spongy mesophilic, through the reflection of infrared radiation within the canopy^28^. Although NDVI is one of the most used indexes, in some situations of abundant and dense vegetation it tends to saturation^29^. Thus, some other indexes were developed, such as SAVI and EVI, which have corrections to reduce atmospheric and soil influences respectively, and consequently, due to the presence of a correction constant, lower values of EVI and SAVI are produced in relation to NDVI^30^.

In this study, EVI and mSAVI_2_ were the most efficient indexes to distinguish vegetation considering different locations and seasonal changes. According to Becerra^31^, EVI is an index developed to optimize the response of the vegetation due to its calculation that considers corrections of distortions of the light reflected by the material suspended in the air, as well as by the ground cover under the vegetation canopy. Laosuwan and Uttaruk^32^ analyzed the efficiency of a model composed by MSAVI_2_ in simulating the variation of tree biomass in Thailand and rated the capacity of this index to verify changes in vegetation over time as efficient. This is possibly due to the fact that mSAVI_2_ is an index that has a positive correlation with the structural changes present in the vegetation because of the minimization of soil influences on the spectral responses of the vegetation^33^.

### Land cover change in regions of the Caatinga biome

Probably, the increase in the conserved Caatinga class in comparison to the change in occupation and land use in Ouricuri and Tauá, occurred due to some fragments of thinned vegetation being in the process of regeneration and ecological succession, and constantly changing its conservation stage due to less anthropic pressure in these areas. In addition, the anthropic action of deforesting fragments of thinned caatinga and herbaceous vegetation for soil preparation and later planting of agricultural crops may have resulted in an increase in the area of bare soil in these regions.

According to the quality scale proposed by Landis and Koch^34^, the Kappa Index values for both Tauá and Ouricuri showed excellent levels of correctness, as well as low values of both errors of omission and commission, with the highest being 32.48% for the herbaceous vegetation class of Ouricuri, while the lowest errors was for the water class. Quite possibly, a factor that may have contributed to the high quality of the classification was the use of images from the dry period of each year of study, which facilitated the distension of classes of vegetation, bare soil and water, since during the dry period of the year, the Caatinga loses its foliage and the agricultural soils are dry. On the other hand, there was a tendency of the classification model to confuse the bare soil class with the herbaceous vegetation, which possibly occurred due to the fact that areas destined for agriculture in these regions remain with exposed soil or with crop residues during the dry period of the year, and the herbaceous vegetation class has essentially small herbaceous plants.

Mapping the coverage of rangelands areas on a temporal and spatial scale is a relevant parameter to provide necessary information for adaptative management in situations of sudden changes in land use^9^, so that quantifying and monitoring the spatial and temporal dynamics of the vegetation cover makes it possible to understand many of the processes of the earth's surface. In the case of the Caatinga management, it is known that it has suffered over time with inadequate methods of use, as well as with the transformation of native forests into agricultural areas^35^. A large part of this biome has been experiencing degradation problems, and the main causes are related to the lack of adequate planning for land use and occupation, and the loss of respect for its characteristics, especially its wealth and biodiversity^36^.

## Conclusion

This study verifies seasonal changes in rangelands using the Wilcoxon t test, and the index's ability to discriminate between different rangelands. The analysis of the spectral responses of Caatinga rangelands is a useful and basic practice for carrying out studies with remote sensing, since it made it possible to distinguish the temporal and spatial variability from the surface reflectance bidirectional obtained in the monitoring sites. The temporal variation in the analysis of the vegetation indexes was a useful tool in identifying seasonal changes in the rangeland, and it can also be useful in identifying the vegetation phenology, as well as in estimating the condition of the vegetation as a feed supply for herds.

In a second step, we perform an image classification in order to produce a tool to monitor the degradation of rangelands. The aim was to study different methods to monitor the rangelands of the Caatinga biome. The image classification model through the Google Earth Engine tool, proved to be effective in verifying the temporal land cover change, indicating that this tool is capable of monitoring information on a large scale, making it possible to identify locations with the vegetation that is most affected and susceptible to degradation, and from that, to know that these regions demand greater government support, as well as intervention with the use of public policies to minimize the damage on the Caatinga biome.
